# Absence of *Plasmodium falciparum* artemisinin resistance gene mutations eleven years after the adoption of artemisinin-based combination therapy in Nigeria

**DOI:** 10.1186/s12936-021-03968-9

**Published:** 2021-11-10

**Authors:** Moses Ikegbunam, Johnson A. Ojo, Kossiwa Kokou, Ugonna Morikwe, Chukwuemeka Nworu, Chibuzo Uba, Charles Esimone, Thirumalaisamy P. Velavan, Olusola Ojurongbe

**Affiliations:** 1 Institute for Tropical Medicine, Tübingen, Germany; 2 Department of Pharmaceutical Microbiology and Biotechnology, Nnamdi Azikiwe University, Awka, Nigeria; 3 Molecular Research Foundation for Students and Scientists, Nnamdi Azikiwe University, Awka, Nigeria; 4 Department of Medical Microbiology & Parasitology, Ladoke Akintola University of Technology, Ogbomoso, Nigeria; 5 Department of Pharmacology and Toxicology, University of Nigeria, Nsukka, Nigeria; 6 Faculty of Medicine, Duy Tan University, Da Nang, Vietnam

**Keywords:** Malaria, Plasmodium falciparum, Drug resistance, Artemisinin, Pfkelch13, Nigeria

## Abstract

**Background:**

The occurrence of artemisinin resistance (ART)-associated polymorphism of *Plasmodium falciparum* K13-propeller (*pfk13*) gene before and after the introduction of artemisinin-based combination therapy (ACT) in two regions of Nigeria was investigated in this study. Regular surveillance is necessary to make a definite conclusion on the emergence and pattern of possible resistance to ART.

**Methods:**

This cross-sectional study was carried out in the Southwestern and Southeastern geopolitical zones of Nigeria. A total of 150, 217, and 475 participants were enrolled for the study in the Southwest (2004_Group A), Southwest (2015_Group B), and southeast (2015_Group C), respectively. Blood samples were collected from the study participants for DNA extraction and a nested PCR for *P. falciparum* identification. Samples that were positive for *P. falciparum* were genotyped for the *pfk13* gene using the Sanger sequencing method. The single nucleotide polymorphisms were analysed using the Bioedit software.

**Results:**

A total of 116, 125, and 83 samples were positive for *P. falciparum,* respectively for the samples collected from the Southwest (2004 and 2015) and southeast (2015). Parasite DNA samples collected from febrile children in 2004 (Group A; n = 71) and 2015 (Group B; n = 73) in Osogbo Western Nigeria and 2015_Group C (n = 36) in southeast Nigeria were sequenced successfully. This study did not observe mutations associated with the in vitro resistance in southeast Asia, such as Y493H, R539T, I543T, and C580Y. Two new polymorphisms V520A and V581I were observed in two samples collected in Osogbo, Southwest Nigeria. These two mutations occurred in the year 2004 (Group A) before the introduction of ACT. Six mutations were identified in 17% of the samples collected in southeast Nigeria. One of these mutations (D547G) was non-synonymous, while the remaining (V510V, R515R, Q613Q, E688E, and N458N) were synonymous. Also, one (2%) heterozygote allele was identified at codon 458 in the 2015 (Group C) samples.

**Conclusions:**

None of the mutations observed in this study were previously validated to be associated with ART resistance. These results, therefore, suggest that artemisinin is likely to remain highly effective in treating malaria in the study areas that are malarious zone.

## Background

Implementing artemisinin-based combination therapy (ACT) as first-line chemotherapy for uncomplicated malaria treatment has led to a 10% reduction in the 1% symptomatic individuals that may progress to fatal severe malaria [1]. Considerable progress was made in the control of malaria between 2000 and 2015, which was attributed to the massive rollout of mosquito nets, effective anti-malarial drugs, and the use of indoor residual spraying of insecticides resulting in the reduction of global malaria mortality and incidence rates by 62 and 41%, respectively [2]. This gain in malaria control is being threatened by the emergence of *Plasmodium falciparum* parasites resistant to artemisinin and its derivatives reported in Southeast Asia.

Artemisinin derivatives are effective fast-acting anti-malarial drugs with a short half-life, resulting in a quick elimination from the human body [3]. Therefore, it was recommended that artemisinin be combined with other structurally different anti-malarial drugs to prevent the likelihood of parasite recovery following a sub-therapeutic blood level. The partner drug is more slowly eliminated and provides complete protection from the development of resistance to the artemisinin component of the combination with an assumption of no high-level resistance to the partner drug [4]. However, artemisinin monotherapies are still available as an over-the-counter medication in countries, where malaria is endemic, thereby increasing the likelihood of solo exposure and the development of resistance when any surviving parasites encounters sub-therapeutic levels [4].

There is a documented resistance to ACT, which was considerably enhanced by the continual use of oral artemisinin monotherapy (AMT), despite the current effort in enforcing their replacement with artemisinin-based combinations [5]. This is a significant problem along the Myanmar/Thailand border, where 16.8% of drug outlets and health facilities are still stocking AMT [6]. In 2007, the World Health Organization (WHO) called for the progressive removal of AMT, and in 2017, they called for a halt in its production and marketing [5], but this seems a bit late as the development of resistance to the artemisinin component of ACT has been documented [7]. So far, all treatment failures of ACT have occurred in Southeast Asia, with earlier reports in Western Cambodia [8, 9]. Historically, every resistance to ACT originated from Southeast Asia, but seemed to have not spread from that region. Thus, there is potential for a spread to sub-Saharan Africa, which is particularly problematic considering that malaria is endemic in the area. Resistance to artemisinins uniquely involves developing a dormant state by the asexual ring blood form leading to recrudescence [10]. Artemisinin resistance is characterized by slower clearance of parasitaemia in the first three days of ACT or artemisinin monotherapy treatment. Close surveillance of the emergence and distribution of artemisinin and partner drug resistance is essential to guide public health measures and monitor spread.

Several single mutations in the *pfk13* gene (which encodes the *P. falciparum* Kelch 13 (*pfk13*) propeller domain) identified in clinical isolates from Cambodia are strongly associated with resistance to artemisinins. In an artificially selected artemisinin-resistant parasite line, only mutations in the *pfk13* propeller domain were reported [11]. The high frequency of *pfk13* mutant alleles facilitating longer parasite clearance half-life was first described in Cambodia [11]. *Pfkelch13* is located on chromosome 13 of *P. falciparum*, which bears several point mutations resulting in an amino acid change, the so-called non-synonymous mutations. The 2018 WHO report on artemisinin resistance has outlined some validated K13 mutations associated with slow parasite clearance, including Y493H, R539T, C580Y, F446I, N458Y, M476I, I543T, P553L, and R561H. In clinical studies, two mutations, K189T and E252Q, occurring outside the K13 propeller domain, have been frequently reported [12]. Furthermore, artemisinin resistance has been linked to mutations in few genes other than *pfk13*, which still needs validation.

Due to the unique artemisinin resistance mechanism, resistance determination is assessed by the ring stage Survival Assay (RSA0-3h) (10) in conjunction with polymorphism of *pfk13* [13]. For example, a large-scale study detected the A578S *pfk13* mutant allele in about 5 out of 12 African countries. This allele has not been confirmed to be associated with reduced parasite clearance or increased RSA0-3 h [14]. A large-scale survey in 59 malaria-endemic countries, including the WHO African region, demonstrated no resistance outside Southeast Asia and China [15], even in the Southwest of Nigeria [16]. Therefore, regular surveillance is necessary to determine the emergence and pattern of possible resistance to ART. These facts motivated this study in which we investigated the presence of K13 mutant alleles in parasites collected before and after the introduction of ACT in two distinct geographical regions of Nigeria, where the incidence of malaria and delayed response to ART have been previously reported [17, 18]. The outcome of this study will contribute to the monitoring of the spread of artemisinin resistance in Sub-Saharan Africa.

## Methods

### Study area and sample collection

The study was conducted in Osogbo, Osun State, in the Southwestern part of Nigeria, and Nnewi, Anambra state, in the southeast of Nigeria. Osogbo is the state capital of Osun State, with a landed area of about 835 hectares and a population projection of over 3 million people as of the 2006 population census. The climate in Osogbo is tropical with two seasons; October to February (dry season) and March to July (rainy season). The average daily temperature is 32ºC with a minimum temperature of 19ºC and a maximum temperature of 35.9ºC. Malaria transmission is usually intense in the rainy season in these two study areas. Nnewi is a town in Anambra State with 194,002 people, annual rainfall of about 1.4 m, and lies mainly in the deciduous forest area, spreading towards the grassland belt (National Census, 2006). Malaria is present throughout the year, with a marked increase during the rainy season, usually from April to September.

Blood samples were collected from 367 febrile children (150 in 2004 and 217 in 2015), between the ages 1–12 years attending the Osun State Hospital, Osogbo [19], and from 475 asymptomatic individuals, between the ages of 1–72 years were recruited during a community survey in southeast Nigeria [20]. Two to three drops of blood were spotted onto 3 MM Whatman filter paper (GE Healthcare Ltd., New Jersey, USA) for genomic DNA extraction. DNA was extracted using the QIAamp® DNA Mini Kit (Qiagen, Hilden, Germany). The samples of the year 2004 (Group A) were collected during a chloroquine efficacy study [19]. The DNA samples were stored at −80^0^C at the Institute for Tropical Medicine in Tübingen, Germany. The 2015 (Group B) samples were from a study that evaluated Nigeria's commercially available rapid diagnostic tests. The 2015 (Group C) samples were from a study in southeast Nigeria that analysed *pfcrt* and *pfmdr1* genes in 2015 [20]. The study received ethical approval from the Ethical Review Committee of the Osun State Ministry of Health, Osogbo (OSHREC/PRS/569 T/130) and the Ethics Review Board, University of Nigeria Teaching Hospital, Enugu, South Eastern Nigeria ((approval number: NHREC/05/01/2008B-FWA00002458-IRB00002323).

### Molecular identification and detection of *pfk13* polymorphisms

The *pfk13* gene was amplified using the conventional nested PCR as previously described [11]. Primers K13-1 and K13-4 were used for the primary PCR (1085 bp), while K13-3 and K13-2 were used for the nested PCR (849 bp). One microlitre of DNA was amplified with 1 μM of each primer, 0.2 mM dNTP (Solis Biodyne), 3 mM MgCl_2_, and 2 U Taq DNA polymerase (Solis Biodyne). Amplicons were detected using 2% agarose gel. All PCR products to be sequenced were purified using Exo-SAP-IT (USB, Affymetrix, USA). Subsequently, 1 µl of the purified product was used as a template for direct sequencing using the Big Dye terminator v. 2.0 cycle sequencer, according to the manufacturer's instructions.

### Sequence analysis and statistics

Descriptive analysis was used to summarize the data (frequencies and mean) using GraphPad Prism 6 (Version 6.0). The statistical significance of the mutations before and after ACT introduction was evaluated using Fisher exact test, while the chi-square test was used to compare the mutations in the three different sample groups (A, B and C), and the significant level set at P < 0.05. Sequences were aligned, and SNP detected using Geneious software (version 9.1.5) and Sequencer (Demo version 4). Sample sequences were aligned to a reference sequence of the K13 protein of the *P. falciparum* isolate 3D7 strain Pf3D7_1343700 of the Plasmodb.org database (plasmodb.org using Geneious Prime and screened for the five mutations M476I, Y493H, R539T, I543T and C580Y. Other minor mutations were also screened.

## Results

### Demographic and parasitology characteristics of the study population

A total of 324 positive *Plasmodium* samples (116 and 125 from southwest Nigeria in 2004 and 2015, respectively, while 83 were from southeast Nigeria in 2015) were analysed in this study. The demographic and parasitology data are shown in Table 1. The mean age for the study population was 3.83 ± 3, 3.83 ± 3, and 19.74 ± 0.64 for 2004_Group A, 2015_Group B, and 2015_Group C, respectively. The mean parasite densities were 9,061, 2866.6, and 1698.56 for the respective regions (Table 1).

**Table 1:** Demographic and parasitology picture of the study population

Sites/Year of sampling	2004_Group A	2015_Group B	2015_Group C
Sample size	150	217	475
Mean Age	3.83 ± 3	8 ± 3.04	19.74 ± 0.64
Sex ration M:F	1:0.68	1:1.1	1:0.79
No (%) positive for *P. falciparum*	116 (77)	125 (57.6)	83 (17.47)
Mean Parasite density (cells/ µl)	9061	2866.6	1698.56
No (%) Amplified for *Pfkelch* 13	71(61.21)	73 (33.64)	36 (7.58)

### Prevalence of SNPs in the *k13* propeller gene

In the samples collected in 2004 (Group A; n = 71) and 2015 (Group B; n = 73) from Osogbo Southwest Nigeria, the entire domain of the *pfk13* was successfully sequenced in 144 samples with *P. falciparum* mono-infection. The mutations associated with in vitro resistance in Southeast Asia, such as Y493H, R539T, I543T, and C580Y, were not observed. Two new polymorphisms were seen in Group A, while none was observed in Group B samples. The mutations in Group A samples were found in positions 520 (nucleotide change C to T resulting in a change in amino acid from valine to alanine (V520A) and 581 (nucleotide change G to A resulting in an amino acid change from valine to isoleucine (V581I) (Table 2). In Group C samples, 36 (100%) positive samples were successfully sequenced. Eighty-three percent of the successfully sequenced samples from Group C had the wild-type allele, while 17% (6/36) had six different mutant alleles (V510V, R515R, Q613Q, E688E, D547G, and N458N). One of these mutations, D547G, is a non-synonymous mutation. This mutation occurred at codon 547, where aspartic acid was changed to glycine. The mutation has not been previously associated with reduced parasite clearance. None of the previously described mutations associated with artemisinin resistance in Southeast Asia regions were detected in the samples. Also, one sample (2%) had a heterozygote allele at codon 458, Table 2. While there was no difference in the prevalence of mutations observed in Group A and B (p = 0.4667), prevalence observed in Group B and C present a significant difference (p = 0.0070).

**Table 2:** Frequency of polymorphisms in *pfk13* gene in *Plasmodium* isolates in different geographical areas in Nigeria

Mutation	2004_Group A (n = 71)		2015 Group_B (n = 73)		2015 Group_C (n = 36)	
	Wild-type n (%)	Mutants n (%)	Wild-type n (%)	Mutants n (%)	Wild-type n (%)	Mutants n (%)
V510V	71 (100)	0 (0)	73 (100)	0 (0)	35 (97.2)	1 (2.8)
R515R	71 (100)	0 (0)	73 (100)	0 (0)	35 (97.2)	1 (2.8)
V520A	70 (98.6)	1 (1.4)	73 (100)	0 (0)	36 (100)	0 (0)
V581I	70 (98.6)	1 (1.4)	73 (100)	0 (0)	36 (100)	0 (0)
D547G	71 (100)	0 (0)	73 (100)	0 (0)	36 (97.2)	1 (2.8)
Q613Q	71 (100)	0 (0)	73 (100)	0 (0)	35 (97.2)	1 (2.8)
E688E	71 (100)	0 (0)	73 (100)	0 (0)	35 (97.2)	1 (2.8)
N458N	71 (100)	0 (0)	73 (100)	0 (0)	35 (92.2)	1 (2.8)

Group A vs Group B (p = 0.4667), Group B Vs Group C ( p = 0.0070), Group A Vs Group C (p = 0.1319), Group A Vs Group B, Vs Group C ( p = 0.0052)

## Discussion

The use of molecular markers such as pfk13 is crucial for drug-resistant surveillance in malaria control programs to find mutations and make proper policy adjustments. In Nigeria, artemisinin-based drugs were introduced as the first-line treatment for malaria in 2005, and to date, resistance has not been confirmed. Artemether-lumefantrine is the first line anti-malarial and the most commonly used ACT in Nigeria. Other commonly used ACT medicines include amodiaquine-artesunate and dihydroartemisinin-piperaquine [21]. This study observed that none of the sequenced parasites had any mutations in the *pfk13* propeller domain associated with artemisinin resistance. The study showed no difference in the prevalence of the mutations before and after the introduction of ACT (Group A and Group B), but showed a significant difference in the two different geographical sites (Group B and Group C). This could be due high use of ACT in southeast Nigeria as shown in a study which revealed that more than 95.8% of the total prescription of anti-malarials in a teaching hospital were artemisinin-based combinations [22], whereas such coverage was not seen in the southwest [23]. It could also be possible that the use of ACT drugs in Nigeria are more with adults than children. A study in southwest Nigeria on the utilization of the current national anti-malarial treatment guidelines among doctors showed that children are more treated with quinine than ACT in that region [24]. In Southwest Nigeria, two new polymorphisms (V520A and V581I) were detected in the samples collected in 2004, while none was observed in the samples collected in 2015. These two minor mutations (V520A and V581I) were also reported in Uganda and DR Congo during a molecular epidemiology survey of pfk13 mutations [25]. Although the phenotypes of these mutant alleles are yet to be linked to artemisinin resistance, they could potentially contribute to delayed parasite clearance in the future in sub-Saharan Africa; further studies are needed to characterize these alleles.

Samples from southeast Nigeria revealed six mutations in the *pfk13* gene, one of which is a non-synonymous mutation that resulted in a switch from aspartic acid to glycine at amino acid position 547 (D547G). There has been no previous report of this mutation, and its association to artemisinin resistance or reduced parasite clearance is unknown. This observation warrants a larger sample size investigation since non-synonymous mutations often produces a protein with altered biochemical properties [26]. Interestingly, two (R515R and N458N) of the remaining five synonymous mutations have been previously observed at very low prevalence in Southeast Asia**,** where its non-synonymous components (R515K and N458Y) were reported [16, 27]. The current observation in this study shows that the study sites are already under pressure. A previous study has shown that the time in hours (h) needed for the parasite density to decline by 50% (PC^1/2^) when R515K and N458Y mutations are present are 5.3 and 7.5 h, respectively. This indicates that N458Y mutation is associated with slow parasite clearance of ACT since PC^1/2^ below 5.5 h results in accelerated ring-form clearance [28]. Two other mutations (V510V and Q613E) recorded in Southeast Nigeria have been previously reported in African countries [16, 28]. Like all mutations observed in Africa, both mutations have not been associated with drug resistance or slow parasite clearance. The general conclusion is that *pfk13* gene mutation is diverse but rare in Africa, showing no evidence for selection even where ACT has been intensively used. No previously detected K13 propeller mutant allele, shown to be associated with reduced parasite clearance or ring survival (the RSA0–3 h phenotype) in Southeast Asia, was detected in this study. The most detected mutation in sub-Saharan Africa has been the A578S, seen in five countries [14, 29] but not in this study. Further, studies demonstrated that parasites possessing this mutant allele in Uganda were sensitive to dihydroartemisinin [30]. Recently, pfk13 non-synonymous mutations associated with delayed parasite clearance in Southeast Asia were detected in 4.5% of isolates collected in 2019 in Rwanda [31]. However, none of the variant parasites showed delayed parasite clearance. It is still difficult to draw a specific conclusion as the outcome could be attributed to the partner drug being very effective or possible partial immunity contributing to parasite elimination.

In a study among children under five years, three artemisinin-based combinations evaluated were still effective ten years after their adoption as first-line therapy for uncomplicated malaria in Nigeria [32]. However, the emergence of D547G mutant alleles in this study or the M579I [33] and A578S mutant allele [14] in Africa's other endemic communities could be a threat to the continued effectiveness of ACT. Unfortunately, this study could not confirm the observed mutations' clinical correlate to artemisinin, a critical requirement in real-time resistance assessment, therefore, further studies focusing on the clinical usefulness of these mutations are recommended.

## Conclusion

This study shed more light on the prevalence of *pfk13* gene in Nigeria. A low prevalence of *pfk13* mutations that are not associated with artemisinin resistance in Southeast Asia was reported. Of interest is the detection of one non-synonymous *pfk13* mutation. Also, there was no difference in the prevalence of mutations before and after the introduction of ACT. However, there was a significant difference in the emergence of mutations in the two geographical regions of Nigeria investigated in the study. Continuous large-scale genomic surveillance of *pfk13* gene mutations is needed to track and monitor parasite responses to ACT in Nigeria.

## Data Availability

The datasets during and/or analysed during the current study are available from the corresponding author on reasonable request.
